# A global model of the response of tropical and sub-tropical forest biodiversity to anthropogenic pressures

**DOI:** 10.1098/rspb.2014.1371

**Published:** 2014-10-07

**Authors:** Tim Newbold, Lawrence N. Hudson, Helen R. P. Phillips, Samantha L. L. Hill, Sara Contu, Igor Lysenko, Abigayil Blandon, Stuart H. M. Butchart, Hollie L. Booth, Julie Day, Adriana De Palma, Michelle L. K. Harrison, Lucinda Kirkpatrick, Edwin Pynegar, Alexandra Robinson, Jake Simpson, Georgina M. Mace, Jörn P. W. Scharlemann, Andy Purvis

**Affiliations:** 1 United Nations Environment Programme World Conservation Monitoring Centre, 219 Huntingdon Road, Cambridge CB3 0DL, UK; 2 Computational Science Laboratory, Microsoft Research Cambridge, 21 Station Road, Cambridge CB1 2FB, UK; 3 Department of Life Sciences, Imperial College London, Silwood Park, Berkshire SL5 7PY, UK; 4 Department of Life Sciences, Natural History Museum, Cromwell Road, London SW7 5BD, UK; 5 BirdLife International, Welbrook Court, Cambridge CB3 0NA, UK; 6 Department of Genetics, Evolution and Environment, Centre for Biodiversity and Environment Research, University College London, Gower Street, London WC1E 6BT, UK; 7 School of Life Sciences, University of Sussex, Falmer, Brighton BN1 9QG, UK

**Keywords:** biodiversity, land-use change, sub-tropical forest, synthetic model, tropical forest

## Abstract

Habitat loss and degradation, driven largely by agricultural expansion and intensification, present the greatest immediate threat to biodiversity. Tropical forests harbour among the highest levels of terrestrial species diversity and are likely to experience rapid land-use change in the coming decades. Synthetic analyses of observed responses of species are useful for quantifying how land use affects biodiversity and for predicting outcomes under land-use scenarios. Previous applications of this approach have typically focused on individual taxonomic groups, analysing the average response of the whole community to changes in land use. Here, we incorporate quantitative remotely sensed data about habitats in, to our knowledge, the first worldwide synthetic analysis of how individual species in four major taxonomic groups—invertebrates, ‘herptiles’ (reptiles and amphibians), mammals and birds—respond to multiple human pressures in tropical and sub-tropical forests. We show significant independent impacts of land use, human vegetation offtake, forest cover and human population density on both occurrence and abundance of species, highlighting the value of analysing multiple explanatory variables simultaneously. Responses differ among the four groups considered, and—within birds and mammals—between habitat specialists and habitat generalists and between narrow-ranged and wide-ranged species.

## 1. Introduction

Habitat loss and degradation, originating mostly from agricultural expansion and intensification, are currently the most common pressures on biodiversity [1]. These pressures affect the structure of local ecological communities and can cause local extinctions of species, which in turn can lead to reduced ecosystem functionality [2,3] and global extinction [1]. The growing human population and changing consumption patterns are likely to cause continued loss of habitat and intensification of land use into the foreseeable future [4,5].

Not all species respond equally to land-use changes: some species are ubiquitous in anthropogenic habitats, whereas others are entirely absent [6]. Responses to land-use and other environmental gradients may be mediated by the functional traits of species: large, slower-breeding, less-mobile species that are dietary and habitat specialists are typically more vulnerable to land-use change than other species [7–13]. The traits that confer vulnerability to land-use change vary geographically [14,15], with tropical forests containing a high proportion of species having traits likely to render them vulnerable to land-use change [13,16]. Tropical forests are predicted to experience among the greatest rates of natural vegetation loss in the near future [17].

The response of species to land-use change can be modelled in three main ways. First, species–area relationships relate loss in the number of species to loss in the area of natural habitat [18]; such models can be applied relatively easily at a global scale, but tend to assume a single relationship between the area of remaining natural habitat and number of species persisting, making it difficult to analyse different responses by different species or to account for non-equilibrium conditions.

Second, species distribution models correlate the current distribution of species to habitat and climate data, and then use these relationships to project the consequences of habitat and climate changes [19]. By capturing individual species' habitat requirements, these models can make detailed, spatially explicit and taxon-specific predictions of range loss, but the data requirements are large and comprehensive data are lacking for many parts of the world and for most taxa [20].

Finally, empirical data from individual studies can be pooled in order to develop synthetic statistical models of the relationship between land-use changes and local occurrence or abundance of species [21,22]; this is the approach we take in this paper. Synthetic analyses take advantage of the widespread availability of multi-species occurrence and abundance data at different sites, often in different land-use types and land-use intensities. Such data can offer a relatively good representation of different taxonomic groups, including traditionally under-represented groups such as invertebrates (see also the electronic supplementary material, table S1). Previous applications of this approach have classified land use into discrete categories based on the description of the habitat given in the source paper [21–23] and have tended to focus on individual taxonomic groups (e.g. [12], but see e.g. [22]). Furthermore, because such studies have usually analysed the effect sizes seen in the source papers rather than the underlying data, they have generally analysed the average response of the whole community rather than the response of individual species, precluding any consideration of different responses among taxonomic or ecological groups [21–23].

Using data collated as part of the projecting responses of ecological diversity in changing terrestrial systems (PREDICTS) project (www.predicts.org.uk), we present an analysis of individual species' responses to land-use and land-use intensity, throughout the world's tropical and sub-tropical forests, of nearly 4000 taxa in four major taxonomic groups: invertebrates, ‘herptiles’ (reptiles and amphibians), mammals and birds. In order to understand changes in community composition and which species are being affected most by land-use change, we also consider differences in responses within these taxonomic groups, between habitat specialists and generalists and between wide-ranging and narrow-ranging species. This is, to our knowledge, the first study to relate differences in responses to human perturbations among and within different taxonomic groups, and the first broad-scale synthetic study to use remotely sensed, and thus globally consistent, data on human land use and other drivers.

## 2. Material and methods

### (a) Study area

**Figure 1.**
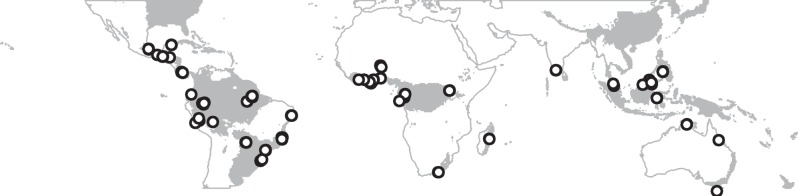
Sites with data used in the models of species occurrence and abundance (circles). Grey shaded areas are those defined as being tropical or sub-tropical forest according to the BIOME model [24].

We focus on studies conducted within forest biomes—i.e. potentially forested areas according to the BIOME model [24] as implemented in the IMAGE model [25]—in tropical and sub-tropical regions of the world (40° S to 40° N) (figure 1).

### (b) Abundance data

Data on the abundance of individual species were gathered from sources identified using a combination of Web of Science (http://wok.mimas.ac.uk) searches, opportunistic surveys of the conservation and applied ecology literature (electronic supplementary material, table S2), and surveys of published meta-analyses of responses of biodiversity to land-use change [22,26,27]. The vast majority of the papers considered implied that the authors attempted to sample all species found within specified taxonomic groups. As with any meta-analysis or synthetic analysis of this kind, not all species will have been sampled during the original surveys. It is likely that undetected species tended to be the rarer species; if the rarer species are also the most sensitive to land-use change, our results will be conservative. For most of the sources, we also obtained additional data from papers' authors, including precise coordinates and plot-specific abundance data (electronic supplementary material, appendix S1). Criteria for including studies were: (i) that the data were collected since 2000 (the earliest year for which the remotely sensed data used here are available); (ii) that the paper contained reported measures of abundance, community composition or diversity from multiple sites at differing levels of human pressures; and (iii) that the coordinates of the sites sampled could be acquired. The final dataset came from 42 published papers and one unpublished database from the Centro Agronómico Tropical de Investigación y Enseñanza (figure 1; electronic supplementary material, appendix S1) and contained 609 sites, and 51 541 abundance records (of which 26% were non-zero values) for 3708 taxa (2138 invertebrates; 295 herptiles; 208 mammals and 1067 birds; electronic supplementary material, table S1). The following measures of abundance were included in the analyses: abundance (47 624 records), relative abundance (3528 records), group abundance (171 records), density (96 records) and reporting rate (122 records). For the measures of abundance that are sensitive to sampling effort (abundance and group abundance), we corrected for any within-study differences in sampling effort by assuming that these measures increase in direct proportion to sampling effort.

### (c) Species habitat specialization and geographical range size

In order to analyse differences in how the occurrence and abundance of species respond to land use *within* major taxonomic groups, we included information on species' degree of habitat specialization and geographical range size. For birds, habitat preferences were classified using the International Union for Conservation of Nature Habitats Classification Scheme. We used data from the highest level, which has 14 broad habitat types including ‘forest’ (http://www.iucnredlist.org/technical-documents/classification-schemes/habitats-classification-scheme-ver3). The importance of each habitat used by species is classified as major, suitable, marginal or unknown, based on information in the literature, and reviewed by experts. We considered any species to be a forest specialist if forest habitat was listed as being of ‘major’ importance. For mammals, we were unable to obtain data on specialization to forest habitats specifically, so instead we used the habitat breadth data from the PanTHERIA database [28], which measures the number of habitat layers (above ground, aquatic, fossorial and ground) used by a species. Species were classified as specialists if they had a habitat breadth of one or as generalists otherwise.

To divide species into wide-ranged and narrow-ranged categories, we first estimated each species' total range area by summing the areas of half-degree grid cells with occurrence records in the Global Biodiversity Information Facility (GBIF) database (http://www.gbif.org/); species whose area of occupancy exceeded the median for the broad taxonomic group were classed as wide-ranged and the others as narrow-ranged. This coarse categorization around taxon-specific medians reflects the fact that taxonomic and geographical biases in GBIF occurrence data preclude simple and accurate estimates of range size [20,29]. The GBIF data were used instead of other data sources, because they allowed comparable estimates of range size for all taxonomic groups, including invertebrates.

### (d) Anthropogenic pressure data

Four measures of anthropogenic environmental pressure were considered as potential explanatory variables for differences in the occurrence and abundance of species: the major land-use type, forest cover, removal of vegetation in the 3 years prior to sampling and human population density.

The major land use at each site within each study was classified as primary vegetation (348 sites), secondary vegetation (94), wood plantation (319), cropland (7), pasture (20) or urban (7) (electronic supplementary material, table S3), based on the description of the habitat given in the original paper. To make the models compatible with globally consistent datasets on land use, we used the same classes as used in the Representative Concentration Pathways scenarios [4]. Numbers of sites in cropland, pasture and urban environments were low, rendering our confidence in the modelled inferences about these habitats lower than for natural and plantation forests. The results for these habitats are presented so that there are at least provisional estimates of the impact of all land-use types, but in the Discussion we focus on the results for forested land-use types.

Forest-cover data for the year 2000 were taken from the moderate-resolution imaging spectroradiometer (MODIS) Vegetation Continuous Fields product [30]. Human population density data for the year 2000 were taken from the Global Rural–Urban Mapping Project, adjusted to match United Nations country-level total population values [31].

Removal of vegetation at each site was estimated from values of the normalized difference vegetation index (NDVI) over the 3 years prior to and including the year of the study. NDVI data were taken from MODIS MOD13Q1 (collection 5) composited for 16 days at 250 m spatial resolution [32] using a development version of the MODISTools package in R [33]. We used a linear interpolation of the raw data, after excluding any data with a quality (QA) flag not equal to zero, and calculated the integrated area under the curve from the minimum observed NDVI value within the 3 years (iNDVI). For studies conducted in 2000, 2001 and 2002, we used NDVI data for the 3 years up to and including 2002. The time integration of NDVI was first suggested by Tucker *et al.* [34] and has been used successfully to estimate crop and wood yields [35] and livestock densities [36].

We removed one statistically influential site with high abundances of reptiles and amphibians, which had an unusual combination of being located in primary forest while having high iNDVI, high forest cover and low human population density. Inspection of the raw NDVI values revealed that the high iNDVI estimate was caused by a single abnormally low NDVI estimate, which was almost certainly caused by unflagged cloud contamination or similar data errors.

### (e) Statistical analysis

The abundance data modelled used several different measures, and both the occurrence and abundance values will have been influenced by study-specific methodological details and by species identity. To control for these effects, the responses of species were fitted using mixed-effects models. Only 26% of the abundance records were non-zero. Therefore, we used a two-stage modelling approach [37], modelling separately the occurrence (assuming detection) of species, using generalized linear mixed-effects models (GLMMs) with a binomial error distribution, and (given presence) log-transformed abundance of species, using linear mixed-effects models (LMMs). We did not model abundances using a GLMM with Poisson errors, because our records of abundance included many non-integer values, as a result of the different types of abundance measure reported in the original papers and/or our correction for sampling effort. All analyses were conducted using R v. 2.15.2 [38]. All GLMMs and LMMs were developed using the lme4 R package (v. 0.999375–42) [39]. For the occurrence models, species with non-zero abundance at a site were taken to be present, while species were assumed to be absent if they were not recorded at that site but were recorded at other sites in the same study. The fit of the final models to the data was assessed by calculating *R*^2^_GLMM_ values [40].

For all models, land use, forest cover, iNDVI and human population density were fitted as fixed effects. All two-way interactions between continuous variables and taxonomic group, two-way interactions between pairs of continuous variables and three-way interactions among pairs of continuous variables and taxonomic group were considered; but not interactions between the continuous variables and the habitat classification, owing to the size of the dataset. The best model, in terms of fixed effects, was selected using backward stepwise variable selection [41]. Site, nested within study, was fitted as a random effect in a random-intercept model to account for different measures and methodologies among studies. A random effect describing the taxonomic affiliation of each record was also included, to account for differences among species unrelated to the explanatory variables of interest. Simpler random-effects structures were considered—taxonomic affiliation only, study only and site nested within study only—but the fit to the data was best with the full set of random effects (assessed using Akaike information criterion (AIC) values [42]).

The results of the models might be influenced by phylogenetic non-independence of responses. We estimated the phylogenetic signal in the residuals of the model against a taxonomic tree. The full taxonomic hierarchy for each record was resolved using the Global Names Resolver (http://resolver.globalnames.org/), which provides a fuzzy search of the Catalogue of Life database (http://www.catalogueoflife.org). A match was obtained for 2789 of the 3708 taxa considered. A tree was constructed based on this taxonomic hierarchy, with branch lengths generated using the Grafen method [43], in the ‘ape’ R package (v. 3.0–2) [44]. Pagel's *λ* statistic [45] was calculated for the taxonomic tree using the ‘geiger’ R package (v. 1.3–1) [46]: a strong phylogenetic signal (*λ* markedly higher than zero) would indicate considerable pseudo-replication. To test whether the model residuals showed significant phylogenetic signal, we compared to a *χ*^2^ distribution the difference in the log-likelihoods (multiplied by two) of the *λ* estimate for the taxonomic tree and for a collapsed tree where all species were assumed to be equally related to one another. Many records could not be matched to a known taxon and models with a taxonomically nested random effect were very computationally intensive; therefore, we were unable to further account for phylogeny in the models.

We tested the effect of habitat specialization and geographical range size on responses to environmental variables in separate post hoc analyses, by refitting the minimum adequate model to separate datasets where all species were divided into broad- and narrow-ranged species, or where birds and mammals were divided into forest/habitat specialists and generalists. We sequentially added an additional interaction with habitat specialization or range size to each term in the minimum adequate model. Improvement in model fit with the addition of each taxonomic-group-by-habitat-specialization or taxonomic-group-by-range-size term was assessed using AIC values.

A non-random spatial configuration of sites within studies might lead to spurious modelled responses, given that species abundance and occurrence are likely to show spatial patterns unrelated to the anthropogenic environmental variables considered. To test the potential for such non-independence to bias our results, we tested for spatial autocorrelation in the residuals of the best models, separately for each major taxonomic group and for each study, using Moran's *I* tests as implemented in the ‘spdep’ package in R (v. 0.5–46) [47]. To check that the conclusions of our models were not affected by any spatial autocorrelation detected, we repeated the final models; dropping data from studies in which we detected significant residual spatial autocorrelation.

## 3. Results

### (a) Occurrence

**Figure 2.**
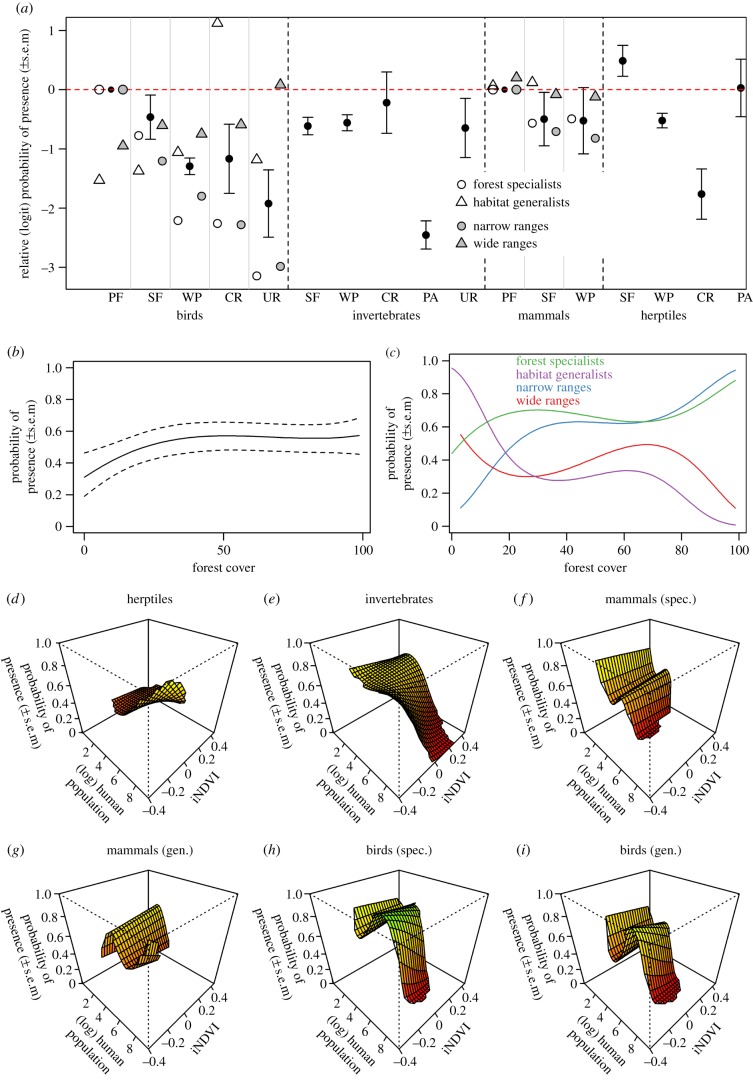
Response of the probability of occurrence of 3708 taxa in tropical forests to land use (*a*), forest cover (*b*,*c*) and the interaction between vegetation removal (iNDVI) and human population density (*d*–*i*). Panel (*a*) shows the relative (logit-transformed) probability of occurrence, relative to the probability of occurrence in primary forest; land-use categories considered were: primary forest (PF), secondary forest (SF), plantation forest (WP), cropland (CR), pasture (PA) and urban (UR); only significant terms are shown. Panels (*b*–*i*) show the absolute (untransformed) probabilities of occurrence, with separate panels for forest/habitat specialists (spec.) and habitat generalists (gen.). Probability of occurrence was estimated using generalized linear mixed-effects models with a binomial error distribution, fitting site nested within study and taxon as random effects. For the response to forest cover, the average response across all species is shown (*b*), as well as the separate responses for habitat specialists and generalists and for narrow-ranged and wide-ranged species (*c*). Error bars (*a*) and dashed lines (*b*) show ±1 s.e. Dashed vertical lines in (*a*) divide the taxonomic groups; grey vertical lines separate the land-use types when taxonomic groups were also divided by habitat specialization and range size.

The probability that species occurred at a site was strongly related to the major land-use type, and this response differed markedly among taxonomic groups (table 1 and figure 2*a*). With the exception of birds in primary forest, narrow-ranged species were less likely than widespread species to occur in all land uses, with the largest differences between narrow- and wide-ranged species seen in urban environments, croplands and plantation forests (ΔAIC = −178; best-fitting model, AIC = 43705; figure 2*a*). Among bird and mammal species, forest specialists were less likely than non-specialists to occur in secondary forest, wood plantation, cropland and urban habitats, but more likely to occur in primary forest (ΔAIC = −262; figure 2*a*).

**Table 1.** Modelled effects of the environmental variables on the probability of occurrence and (given presence) abundance of species. (Terms were sequentially removed in a backward stepwise selection and tested with analysis of variance. Main effects were tested after removing all interaction terms from the model. Significant (*α* < 0.05) terms are italicized. Terms ‘n.a.’ are interaction terms whose inclusion was not supported by preliminary modelling. HPD, human population density.)

term	occurrence		abundance	
	*χ*^2^	*p*	*χ*^2^	*p*
land use : taxonomic group	*219*	<*0.001*	*65.2*	<*0.001*
HPD	*34.8*	<*0.001*	0.849	0.36
forest cover	*7.96*	*0.047*	0.451	0.50
iNDVI	1.01	0.31	*101*	<*0.001*
HPD : forest cover	*27.9*	<*0.001*	0.00	1.0
HPD : iNDVI	*9.39*	*0.025*	1.96	0.58
forest cover : iNDVI	5.34	0.14	<0.001	1.0
HPD : taxonomic group	16.1	0.063	n.a.	n.a.
forest cover : taxonomic group	0.00	1.00	*40.3*	<*0.001*
iNDVI : taxonomic group	n.a.	n.a.	*140*	<*0.001*
HPD : forest cover : taxonomic group	0.00	1.00	*13.3*	*0.0098*
HPD : iNDVI : taxonomic group	0.00	1.00	14.2	0.12
forest cover : iNDVI : taxonomic group	0.00	1.00	*73.8*	<*0.001*

Probability of occurrence varied significantly with forest cover, human population density and iNDVI (table 1). While the major taxonomic groups responded similarly to forest cover (figure 2*b*), human population density and iNDVI, fitting the two-way interactions between the more refined taxonomic classification—which divided birds and mammals into habitat specialists and generalists—and both human population density and forest cover did result in a significant improvement in model fit (ΔAIC = −31.7 and −3.39, respectively). Similarly, dividing species into narrow-and wide-ranging species led to a slight improvement in model fit with respect to forest cover (ΔAIC = −0.30).

All taxonomic groups were slightly more likely to occur with increasing forest cover (figure 2*b*). However, this relationship masked variation among species within taxonomic groups: forest specialist and narrow-ranged bird species were much more likely to occur where forest cover was higher, whereas habitat generalist and wide-ranged bird species were less likely to do so (figure 2*c*).

The probability of occurrence of herptiles was highest at high human population density, but decreased slightly with increasing iNDVI (figure 2*d*). There were insufficient data to divide reptiles and amphibians in the full models, but running simple models of probability of occurrence against human population density (with a cubic polynomial) for reptiles and amphibians separately revealed a U-shaped relationship between human population density and probability of occurrence for reptiles and a monotonically increasing relationship for amphibians (electronic supplementary material, figure S1). Invertebrates were least likely to occur at sites with a combination of high human population density and high iNDVI (figure 2*e*). Occurrence of habitat specialist mammals and birds declined sharply with increasing human population density, and to a lesser extent with increasing iNDVI (figure 2*f*,*h*). Habitat generalist bird species were also generally less likely to occur at higher human population density and to a lesser extent at higher iNDVI, but showed a peak in probability of occurrence at intermediate human population densities (figure 2*i*). Habitat-generalist mammal species were most likely to occur at intermediate human population densities and at higher iNDVI (figure 2*g*).

Land use, forest cover, iNDVI and human population density explained a relatively small amount of the variation in probability of occurrence, after accounting for study- and taxon-level differences (marginal 
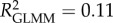
). The residuals of the model of species occurrence showed weak but highly significant phylogenetic signal (*λ* = 0.10; *χ*^2^ test: *p* < 0.001).

### (b) Abundance

**Figure 3.**
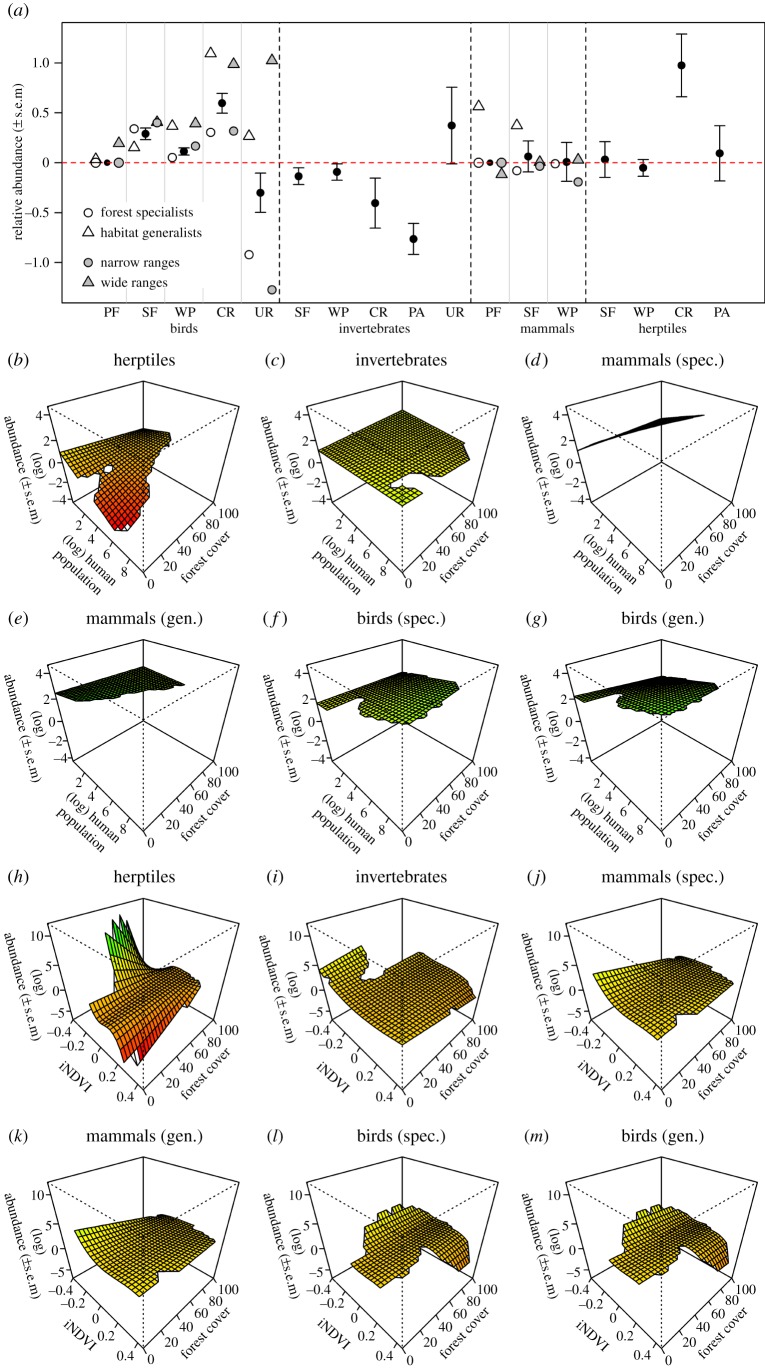
Response of the abundance of 3708 taxa in tropical forests to land use (*a*), the interaction between human population density and forest cover (*b*–*g*), and the interaction between forest cover and vegetation removal (iNDVI; *h*–*m*). Panel (*a*) shows the relative (log-transformed) abundance, relative to the abundance in primary forest; land-use categories considered were: primary forest (PF), secondary forest (SF), plantation forest (WP), cropland (CR), pasture (PA) and urban (UR); only significant terms are shown. Panels (*b*–*m*) show absolute (log-transformed) abundance, with separate panels for forest/habitat specialists (spec.) and habitat generalists (gen.). Log-transformed abundance was modelled using linear mixed-effects models, fitting site nested within study and taxon as random effects. Error bars (*a*) show ±1 s.e. Dashed vertical lines in (*a*) divide the taxonomic groups; grey vertical lines separate the land-use types when taxonomic groups were also divided by habitat specialization and range size.

Species' abundances responded significantly to land-use type, with large differences among taxonomic groups (table 1 and figure 3*a*). Narrow-ranged species tended to be less abundant than widespread species in all land-use types but especially in urban environments, croplands and plantation forests (ΔAIC = −47.3; best-fitting model, AIC = 38145; figure 3*a*). Similarly, among mammals and birds, habitat specialists tended to be less abundant than habitat generalists, especially in urban habitats, croplands and plantation forests (ΔAIC = −15.4; figure 3*a*). With the exception of invertebrates, species present in secondary forest, wood plantation and cropland were more abundant there than in primary forest, whereas even those species that occurred in urban habitats were less abundant there than in primary forest (figure 3*a*).

Abundance varied with forest cover, human population density and iNDVI; all two-way interactions were significant, and different taxonomic groups showed significantly different responses (table 1). Furthermore, wide- and narrow-ranging species showed slightly different responses to forest cover and iNDVI (ΔAIC = −10.0 and −2.70, respectively; electronic supplementary material, figure S2) and, among birds and mammals, habitat specialists and habitat generalists showed different responses to forest cover (ΔAIC = −3.96) and human population density (ΔAIC = −7.09). Herptile abundance declined with increasing human population density, decreasing forest cover and increasing iNDVI (figure 3*b*,*h*). For invertebrates and most mammals and birds, abundance varied very little with forest cover and human population density (figure 3*c*–*g*), although abundance increased slightly with human population density for habitat specialist mammal species (figure 3*d*). Invertebrates, birds and mammals all decreased slightly in abundance with increasing iNDVI (figure 3*i*–*m*).

Land use, forest cover, iNDVI and human population density explained little of the variation in abundance, after accounting for study- and taxon-level differences (marginal 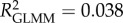). The residuals of the model of species abundance were distributed nearly normally (electronic supplementary material, figure S3) and showed weak, but significant, phylogenetic signal (*λ* = 0.063; *χ*^2^ test: *p* < 0.001).

### (c) Sampled sites

Sampled sites were not distributed evenly with respect to the environmental variables. Sites were located in primary forest (348 sites), secondary forest (94) and wood plantations (319) more often than in cropland (seven sites; of which four for invertebrates, two for herptiles and one for birds), pasture (20 sites; 17 for invertebrates and three for herptiles) and urban habitats (seven sites; six for invertebrates and one for birds) (electronic supplementary material, figure S4). Sites sampled for herptiles were more patchily distributed with respect to human population density, forest cover and iNDVI than the other taxonomic groups (electronic supplementary material, figures S5 and S6). Given that we can place much greater confidence in the results for primary and secondary forest and wood plantation than for cropland, pasture and urban habitats, we will focus on these results in the Discussion.

Variance in occurrence and abundance was greatest among studies, then among taxa and finally among sites within studies (electronic supplementary material, table S4). The residuals of the best model showed significant spatial autocorrelation for more of the studies than would be expected by chance (17.3% and 15.4% for the occurrence and abundance models, respectively; electronic supplementary material, figures S7 and S8). The abundance dataset excluding studies with significant spatial autocorrelation in the model residuals was little more than half the size of the original dataset. Removing these studies and re-running the best models did not change the shape of the fitted responses (electronic supplementary material, figures S9 and S10), although uncertainty increased markedly for the modelled responses of the abundance of taxa which lost many data in this process—herptiles and birds (both specialists and generalists).

## 4. Discussion

Land use has long been recognized as a profound influence on ecological communities [22,48]. Precisely quantifying this influence is an essential step towards ecological sustainability but has been problematic because responses to a given impact can vary among response variables, and both among and within major taxa. Our results give, to the best of our knowledge, the clearest and most precise picture to date of the consistent and profound effect that land use has on ecological communities throughout the world's tropical and sub-tropical forest biome and show that different taxonomic groups, and different types of species within taxonomic groups, respond very differently to land-use change.

Overall, the probability of occurrence of species in all taxonomic groups declined in human-modified habitats, whereas persisting species often increased in abundance. These effects together led to increased dominance by smaller numbers of taxa. However, at the community level, the increases in abundance never compensated for the decreased occurrence of species: a crude measure of total community abundance (the product of relative probability of occurrence and relative abundance of persisting species) ranged from 7.9% (invertebrates in pastures) to 62% (herptiles in secondary forest) of the value in primary forest. We show that the taxa benefiting from land-use change are generally the more geographically widespread species, and the species that are more generalist in terms of habitat use. Modelling the occurrence and abundance of individual species—for, to our knowledge, the first time at this scale—allowed us to show marked differences in species' responses both among and within major taxonomic groups. Using remotely sensed data and other data that are consistent across the study area allowed for a more consistent characterization of the habitat at each site.

The increase in the abundance of birds in plantation forests may be because certain types of resources remain abundant in these habitats allowing the species that use them to persist in large numbers, or perhaps because of retention of the vertical structure of natural vegetation, which can have a strong effect on bird community structure [49]. It is also possible that increased detectability in plantation forests led to some of the reported increases in abundance but is unlikely to be the only explanation given the concomitant decrease in occurrence. Birds were highly sensitive to urban land use, declining markedly in both occurrence and abundance compared with primary forest. This is supported by the finding that bird species declined markedly in their probability of occurrence with increasing human population density, consistent with previous studies of bird species in urban habitats [50–52]. Forest specialists and narrow-ranged species were the most severely impacted, being less than 10% as likely to occur in urban habitats as in primary forest. Although numbers of sites in cropland, pasture and urban habitats were low, the disproportionate impact on forest specialists and narrow-ranged species is suggestive of the biotic homogenization of community composition and warrants further analysis with expanded datasets.

Surprisingly, herptiles (both reptiles and amphibians) were more likely to occur at higher human population density, although sampling of herptiles along the gradient of human population density was patchy. This suggests that open habitats, associated with higher human population density, benefit a greater number of species than do more closed habitats. This interpretation is supported by the higher probability of occurrence of herptile species in secondary forest compared with primary forest. The result might also be explained by increased detectability in open habitats, which is likely to be particularly pronounced for herptiles compared to other taxonomic groups; however, if detectability were solely responsible, one would expect abundance to also increase with human population density, which it did not. The decline in herptile abundance with increasing human population density, increasing vegetation removal and decreasing forest cover suggests that while more open habitats might support a greater number of species, human-dominated habitats have much lower abundances.

Among mammals, habitat specialist and narrow-ranging species had much-reduced probabilities of occurrence in non-primary habitats. Furthermore, habitat specialist mammals were highly sensitive to human population density, with declines of about 70% in probability of occurrence across the gradient of human population densities sampled. The overall negative effect of human population density may be the result of direct effects, such as hunting [53], or indirect effects of, for example, human infrastructure. Previous studies have shown marked declines in the abundance of mammals near to roads [54–56]. The peak in the probability of occurrence of mammal species at intermediate human population densities suggests that at least some species benefit from mild human disturbance.

Most of the variation in the occurrence and abundance of species within studies remained unexplained. Additional, more finely resolved and more accurate habitat information, and information on other factors affecting species, may help to constrain the estimates of occurrence and abundance. Regardless of the quality of the environmental data used in the models, different species are likely to respond differently to anthropogenic disturbances [7–13], in ways that additional trait data might help to explain.

Interactions among species—which we did not consider—are important determinants of the occurrence and abundance of species [57]. In future, the incorporation of interactions into models may allow more accurate predictions of how species respond [58]. We also did not account for the known effects of habitat patch size and fragmentation on occurrence and abundance [59].

Spatial and phylogenetic autocorrelation can bias inferences about the response of species to environmental gradients [60]. We detected residual spatial autocorrelation in a minority of studies considered. Computational limitations prevented the inclusion of spatial autocorrelation alongside the already-complex random-effects structures in our models, but removing data from the affected studies and refitting the models had little effect on the modelled responses. We were also prevented from fully accounting for phylogeny by computational limitations and the lack of a full taxonomic hierarchy for many of the species considered. We detected significant phylogenetic signals in the residuals of our models, suggesting that in the future, given more complete phylogenies and more computational power, the modelling could be improved by better accounting for the relatedness of species. However, the strength of phylogenetic signal in the residuals of the model was low, suggesting that the effects of incorporating phylogeny would be slight.

Overall, the results demonstrate that transformation of habitats for human land use is causing consistent reductions in species richness and changes in abundance, altering ecological communities in tropical and sub-tropical forests around the world. Human-dominated habitats have fewer species than natural habitats. The results add to a growing body of evidence that humans are causing fundamental changes to community structure. Collating published data on species occurrence and abundance opens new opportunities for assessing biodiversity state, and analyses like ours can be expanded to other biomes for which data are available. Using land-use information that follows a widely used classification scheme, as well as globally consistent environmental data, makes these models a strong basis for extrapolating community responses across space and through time, which will be essential for predicting the biodiversity impacts of future changes.

## Supplementary Material

Supplementary tables and figures

## Data accessibility

We do not own the rights to any of the occurrence and abundance data used in the analyses. The data will be published in 2015 as part of the PREDICTS Project database. In the meantime, we will forward any requests for these data to the data owners. The habitat specialization data are available from BirdLife International http://www.birdlife.org.uk/datazone/home and the mammal habitat data from PanTHERIA (http://esapubs.org/archive/ecol/e090/184/).
