# Supplementary material for: A global model of the response of tropical and sub-tropical forest biodiversity to anthropogenic pressures
Source: Proc Biol Sci. 2014 Oct 7;281(1792):20141371. doi: 10.1098/rspb.2014.1371 (PMC4150326; doi:10.1098/rspb.2014.1371)
Supplement: Supplementary tables and figures [file rspb20141371supp1.pdf]

Supplementary Material

Table S1. Numbers of abundance records and studies for each major taxonomic group. Note that some studies considered more than one taxonomic group, hence the total number of studies is greater than the number of source papers quoted in the text.

major taxonomic group	number of abundance records	number of studies
Invertebrates	20111	28
Reptiles & Amphibians	10370	5
Mammals	4928	10
Birds	16132	10
**Total**	51541	53

Table S2. Search terms used to find papers in the Web of Science database (http://wok.mimas.ac.uk), and journals for which recent issues were searched for papers with appropriate data.

**Web of Knowledge search terms:**
"[species] AND [tropic*] AND [primary forest OR mature forest OR intact forest OR old growth forest OR virgin forest OR pristine forest] AND [conversion OR degradation OR land use OR habitat type OR agro-forestry OR secondary forest OR plantation OR cropland OR urban area] AND [diversity OR biodiversity OR richness OR abundance]"
"habitat fragmentation AND abundance AND mammal, bird, amphibian, reptile"
"deforestation AND biodiversity AND mammal, bird, amphibian, reptile"
"Insect* Diversity AND Land Use Change"
"Insect* Diversity AND Tropical Deforest*"
"(species diversity, biodiversity, richness OR abundance) AND (land use OR habitat conversion) AND (pristine, primary, undisturbed OR original)"
"palm oil AND biodiversity"
"cocoa AND biodiversity"
"cacao AND biodiversity"

**journals scanned for papers with relevant data:**
Biodiversity and Conservation
Biological Conservation
Conservation Biology

Animal Conservation

Diversity and Distributions

Insect Conservation and Diversity

Conservation and Biodiversity

Journal of Applied Ecology

Biotropica

Agricultural and Forest Entomology

Appendix S1. List of papers from which abundance data were obtained. For those marked with an *, we obtained additional data from the authors – coordinate data, or site-level data where the paper presented averages for land-use types. For all other papers, all data necessary for this analysis were presented in the paper.

1 Alcala, E. L., Alcala, A. C. & Dolino, C. N. 2004 Amphibians and reptiles in tropical rainforest fragments on Negros Island, the Philippines. *Environ. Conserv.* **31**, 254–261. (doi:10.1017/S0376892904001407)

2 Andersen, A. N., Ludwig, J. A., Lowe, L. M. & Rentz, D. C. F. 2001 Grasshopper biodiversity and bioindicators in Australian tropical savannas: Responses to disturbance in Kakadu National Park. *Austral Ecol.* **26**, 213–222. (doi:10.1046/j.1442-9993.2001.01106.x)

3 Arellano, L., León-Cortés, J. L. & Halffter, G. 2008 Response of dung beetle assemblages to landscape structure in remnant natural and modified habitats in southern Mexico. *Insect Conserv. Divers.* **1**, 253–262. (doi:10.1111/j.1752-4598.2008.00033.x)

4 Barlow, J., Mestre, L. A. M., Gardner, T. A. & Peres, C. A. 2007 The value of primary, secondary and plantation forests for Amazonian birds. *Biol. Conserv.* **136**, 212–231. (doi:10.1016/j.biocon.2006.11.021)

5 Basset, Y. et al. 2008 Changes in arthropod assemblages along a wide gradient of disturbance in Gabon. *Conserv. Biol.* **22**, 1552–63. (doi:10.1111/j.1523-1739.2008.01017.x)

6 Bonham, K. J., Mesibov, R. & Bashford, R. 2002 Diversity and abundance of some ground-dwelling invertebrates in plantation vs. native forests in Tasmania, Australia. *For. Ecol. Manage.* **158**, 237–247. (doi:10.1016/S0378-1127(00)00717-9)

7 Bouyer, J., Sana, Y., Samandoulgou, Y., Cesar, J., Guerrini, L., Kabore-Zoungrana, C. & Dulieu, D. 2007 Identification of ecological indicators for monitoring ecosystem health in the trans-boundary W Regional park: A pilot study. *Biol. Conserv.* **138**, 73–88. (doi:10.1016/j.biocon.2007.04.001)

8 Cáceres, N. C., Nápoli, R. P., Casella, J. & Hannibal, W. 2010 Mammals in a fragmented savannah landscape in south-western Brazil. *J. Nat. Hist.* **44**, 491–512. (doi:10.1080/00222930903477768)

9 Chiarello, A. G. 1999 Effects of fragmentation of the Atlantic forest on mammal communities in south-eastern Brazil. *Biol. Conserv.* **89**, 71–82.

10 Davis, A. L. V & Philips, T. K. 2005 Effect of deforestation on a Southwest Ghana dung beetle assemblage (Coleoptera : Scarabaeidae) at the periphery of Ankasa

conservation area. *Environ. Entomol.* **34**, 1081–1088. (doi:10.1603/0046-225X(2005)034)

11 Fabricius, C., Burger, M. & Hockey, P. A. R. 2003 Comparing biodiversity between protected areas and adjacent rangeland in xeric succulent thicket, South Africa: arthropods and reptiles. *J. Appl. Ecol.* **40**, 392–403. (doi:10.1046/j.1365-2664.2003.00793.x)

12 Filgueiras, B. K. C., Iannuzzi, L. & Leal, I. R. 2011 Habitat fragmentation alters the structure of dung beetle communities in the Atlantic Forest. *Biol. Conserv.* **144**, 362–369. (doi:10.1016/j.biocon.2010.09.013)

13 Gardner, T. A., Hernández, M. I. M., Barlow, J. & Peres, C. A. 2008 Understanding the biodiversity consequences of habitat change: the value of secondary and plantation forests for neotropical dung beetles. *J. Appl. Ecol.* **45**, 883–893. (doi:10.1111/j.1365-2664.2008.01454.x)

14 Gardner, T. A., Ribeiro-Júnior, M. A., Barlow, J., Ávila-Pires, T. C. S., Hoogmoed, M. S. & Peres, C. A. 2007 The value of primary, secondary, and plantation forests for a Neotropical herpetofauna. *Conserv. Biol.* **21**, 775–87. (doi:10.1111/j.1523-1739.2007.00659.x)

15 Gottschalk, M. S., De Toni, D. C., Valente, V. L. S. & Hofmann, P. R. P. 2007 Changes in Brazilian Drosophilidae (Diptera) assemblages across an urbanization gradient. *Neotrop. Entomol.* **36**, 848–862.

16 Hawes, J., da Silva Motta, C., Overal, W. L., Barlow, J., Gardner, T. A. & Peres, C. A. 2009 Diversity and composition of Amazonian moths in primary, secondary and plantation forests. *J. Trop. Ecol.* **25**, 281–300. (doi:10.1017/S0266467409006038)

17 Johnson, M. F., Gómez, A. & Pinedo-Vasquez, M. 2008 Land use and mosquito diversity in the Peruvian Amazon. *J. Med. Entomol.* **45**, 1023–1030. (doi:10.1603/0022-2585(2008)45)

18 Kapoor, V. 2007 Effects of rainforest fragmentation and shade-coffee plantations on spider communities in the Western Ghats, India. *J. Insect Conserv.* **12**, 53–68. (doi:10.1007/s10841-006-9062-5)

19 Kone, M., Konate, S., Yeo, K., Kouassi, P. K. & Linsenmair, K. E. 2010 Diversity and abundance of terrestrial ants along a gradient of land use intensification in a transitional forest-savannah zone of Côte d'Ivoire. *J. Appl. Biosci.* **29**, 1809–1827.

20 Matsumoto, T., Itioka, T., Yamane, S. & Momose, K. 2009 Traditional land use associated with swidden agriculture changes encounter rates of the top predator, the army ant, in Southeast Asian tropical rain forests. *Biodivers. Conserv.* **18**, 3139–3151. (doi:10.1007/s10531-009-9632-4)

21 Mena, J. L. & Medellín, R. A. 2010 Small mammal assemblages in a disturbed tropical landscape at Pozuzo, Peru. *Mamm. Biol.* **75**, 83–91. (doi:10.1016/j.mambio.2009.08.006)

22 Navarrete, D. & Halffter, G. 2008 Dung beetle (Coleoptera: Scarabaeidae: Scarabaeinae) diversity in continuous forest, forest fragments and cattle pastures in a landscape of Chiapas, Mexico: the effects of anthropogenic changes. *Biodivers. Conserv.* **17**, 2869–2898. (doi:10.1007/s10531-008-9402-8)

23 Nyeko, P. 2009 Dung beetle assemblages and seasonality in primary forest and forest fragments on agricultural landscapes in Budongo, Uganda. *Biotropica* **41**, 476–484. (doi:10.1111/j.1744-7429.2009.00499.x)

24 O'Dea, N. & Whittaker, R. J. 2007 How resilient are Andean montane forest bird communities to habitat degradation? *Biodivers. Conserv.* **16**, 1131–1159. (doi:10.1007/s10531-006-9095-9)

25 Parry, L., Barlow, J. & Peres, C. A. 2009 Hunting for sustainability in tropical secondary forests. *Conserv. Biol.* **23**, 1270–1280. (doi:10.1111/j.1523-1739.2009.01224.x)

26 Slade, E. M., Mann, D. J. & Lewis, O. T. 2011 Biodiversity and ecosystem function of tropical forest dung beetles under contrasting logging regimes. *Biol. Conserv.* **144**, 166–174. (doi:10.1016/j.biocon.2010.08.011)

27 Soh, M. C. K., Sodhi, N. S. & Lim, S. L. H. 2006 High sensitivity of montane bird communities to habitat disturbance in Peninsular Malaysia. *Biol. Conserv.* **129**, 149–166. (doi:10.1016/j.biocon.2005.10.030)

28 Vallan, D. 2002 Effects of anthropogenic environmental changes on amphibian diversity in the rain forests of eastern Madagascar. *J. Trop. Ecol.* **18**, 725–742. (doi:10.1017/S026646740200247X)

29 Woinarski, J. C. Z. & Ash, A. J. 2002 Responses of vertebrates to pastoralism, military land use and landscape position in an Australian tropical savanna. *Austral Ecol.* **27**, 311–323. (doi:10.1046/j.1442-9993.2002.01182.x)

30 Kessler, M. et al. 2009 Alpha and beta diversity of plants and animals along a tropical land-use gradient. *Ecol. Appl.* **19**, 2142–2156.

31 Vergara, C. H. & Badano, E. I. 2009 Pollinator diversity increases fruit production in Mexican coffee plantations: The importance of rustic management systems. *Agric. Ecosyst. Environ.* **129**, 117–123. (doi:10.1016/j.agee.2008.08.001)

32 Lachat, T., Attignon, S., Djego, J., Goergen, G., Nagel, P., Sinsin, B. & Peveling, R. 2006 Arthropod diversity in Lama forest reserve (South Benin), a mosaic of natural, degraded and plantation forests. *Biodivers. Conserv.* **15**, 3–23. (doi:10.1007/s10531-004-1234-6)

33 Wunderle, J. M., Henriques, L. M. P. & Willig, M. R. 2006 Short-term responses of birds to forest gaps and understory: an assessment of reduced-impact logging in a lowland Amazon Forest. *Biotropica* **38**, 235–255. (doi:10.1111/j.1744-7429.2006.00138.x)

34 Bragagnolo, C., Nogueira, A. A., Pinto-da-Rocha, R. & Pardini, R. 2007 Harvestmen in an Atlantic forest fragmented landscape: Evaluating assemblage response to habitat quality and quantity. *Biol. Conserv.* **139**, 389–400. (doi:10.1016/j.biocon.2007.07.008)

35 MacSwiney G., M. C., Vilchis L., P., Clarke, F. M. & Racey, P. A. 2007 The importance of cenotes in conserving bat assemblages in the Yucatan, Mexico. *Biol. Conserv.* **136**, 499–509. (doi:10.1016/j.biocon.2006.12.021)

36 Presley, S. J., Willig, M. R., Wunderle Jr., J. M. & Saldanha, L. N. 2007 Effects of reduced-impact logging and forest physiognomy on bat populations of lowland Amazonian forest. *J. Appl. Ecol.* **45**, 14–25. (doi:10.1111/j.1365-2664.2007.01373.x)

37 Wells, K., Kalko, E. K. V., Lakim, M. B. & Pfeiffer, M. 2007 Effects of rain forest logging on species richness and assemblage composition of small mammals in Southeast Asia. *J. Biogeogr.* **34**, 1087–1099. (doi:10.1111/j.1365-2699.2006.01677.x)

38 Willig, M. R., Presley, S. J., Bloch, C. P., Hice, C. L., Yanoviak, S. P., Díaz, M. M., Chauca, L. A., Pacheco, V. & Weaver, S. C. 2007 Phyllostomid bats of lowland Amazonia: effects of habitat alteration on abundance. *Biotropica* **39**, 737–746. (doi:10.1111/j.1744-7429.2007.00322.x)

39 Horgan, F. G. 2009 Invasion and retreat: shifting assemblages of dung beetles amidst changing agricultural landscapes in central Peru. *Biodivers. Conserv.* **18**, 3519–3541. (doi:10.1007/s10531-009-9658-7)

40 Sheldon, F. H., Styring, A. & Hosner, P. A. 2010 Bird species richness in a Bornean exotic tree plantation: A long-term perspective. *Biol. Conserv.* **143**, 399–407. (doi:10.1016/j.biocon.2009.11.004)

41 Phalan, B., Onial, M., Balmford, A. & Green, R. E. 2011 Reconciling food production and biodiversity conservation: land sharing and land sparing compared. *Science (80-.).* **333**, 1289–1291. (doi:10.1126/science.1208742)
42 Henschel, P. 2008 The conservation biology of the leopard Panthera pardus in Gabon: Status, threats and strategies for conservation.

Table S3. The classification scheme used to assign sites to land-use and land-use-intensity classes. Note that land-use-intensity was not considered in the current study.

Land-use	Description
Primary forest	Forest composed of native vegetation, which is not known to have been destroyed during historical times
Secondary Vegetation	Previously destroyed vegetation recovering to natural state rather than being managed to maintain it in a non-natural state
Plantation forest	Managed plantations for timber, fruit, coffee, oil-palm, rubber or any other woody crop
Cropland	Arable farmland
Pasture	Grazed pasture
Urban	Villages, suburban areas or cities, including managed green spaces

Table S4. Variance in occurrence and abundance explained by the random effects considered in the models.

Random effect	Variance in occurrence explained	Variance in abundance explained
Study	2.8	9.01
Site within study	0.21	0.049
Taxon	1.87	0.9

Figure S1. Relationship between human population density and the probability of occurrence of reptiles (red line), amphibians (green lines), and reptiles and amphibians together (black line), based on models using only human population density (with a cubic polynomial) as a fixed effect.

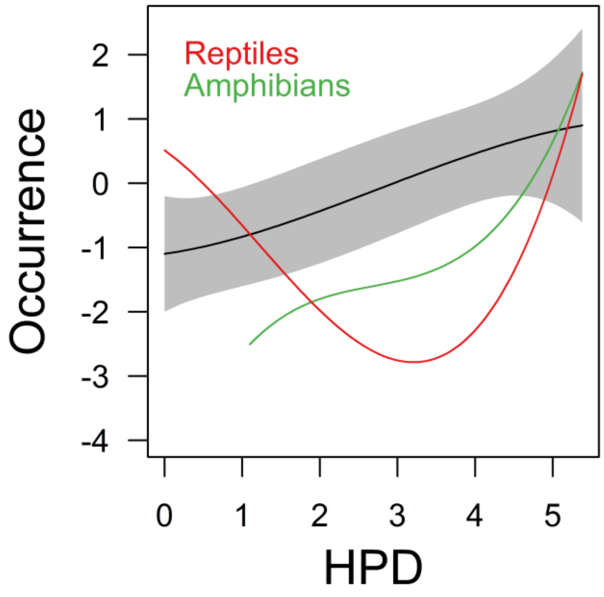

Figure S2. Response of the abundance of different major taxonomic groups, divided into widespread (wide) and narrow-ranging (narrow) species, to the interaction between forest cover and vegetation removal (iNDVI). Values are absolute (log-transformed) abundance. Log-transformed abundance was modelled using linear mixed-effects models, fitting site nested within study and taxon as random effects.

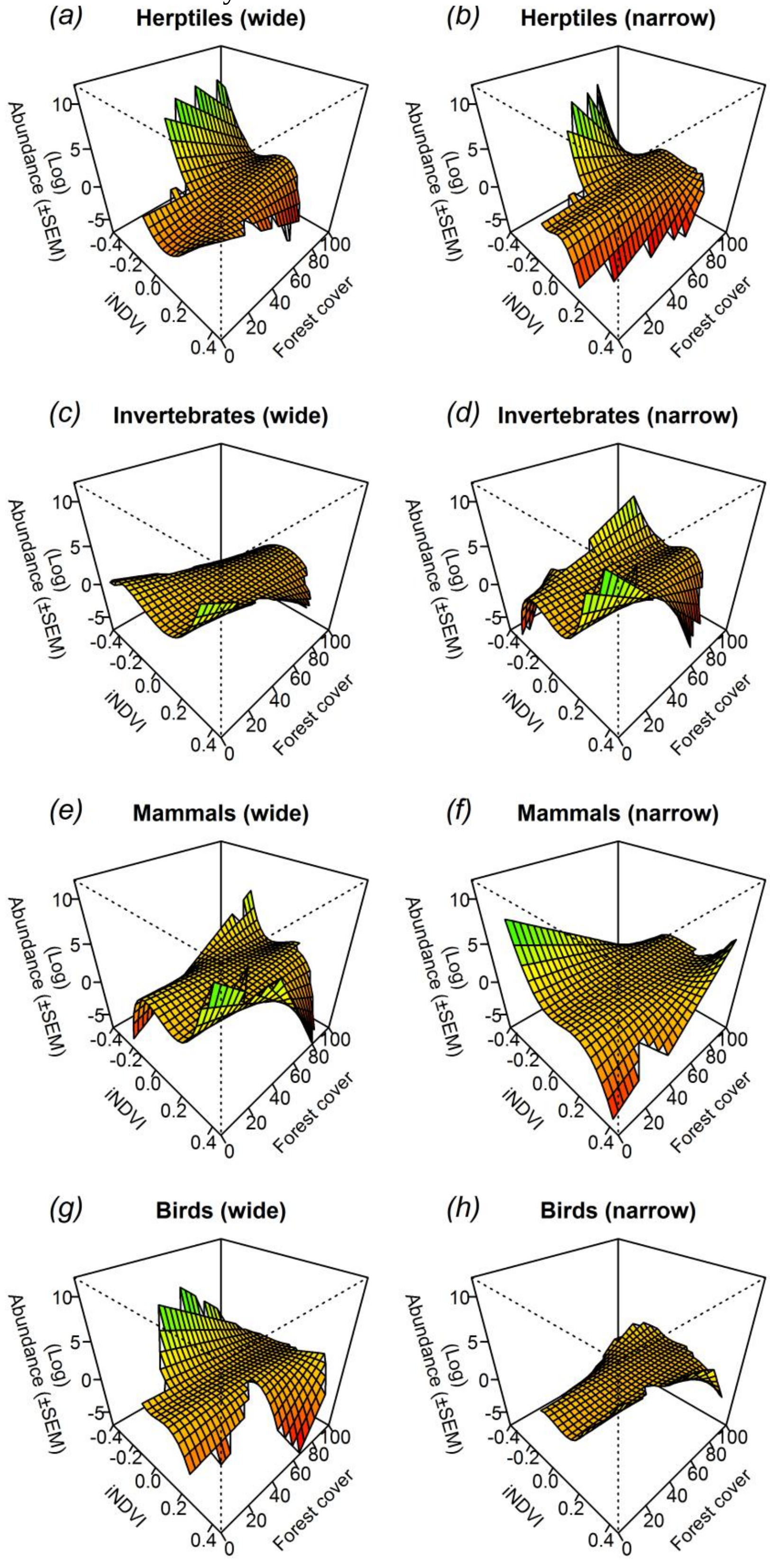

Figure S3. Q-Q plot to test for normality in the distribution of residuals from the final model of log-transformed abundance. Kolmogorov-Smirnov test: D = 0.114, P < 0.001.

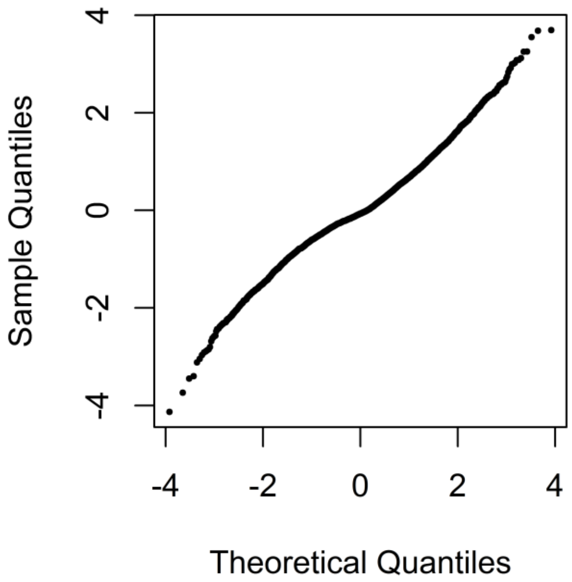

Figure S4. The representation of different the land-use classes by studies (red bars) and by sampled sites (blue bars), for the major taxonomic groups considered. PF = primary forest; SF = secondary forest; WP = plantation forest; CR = cropland; PA = pasture; UR = urban. See the main text for details of the land-use classification.

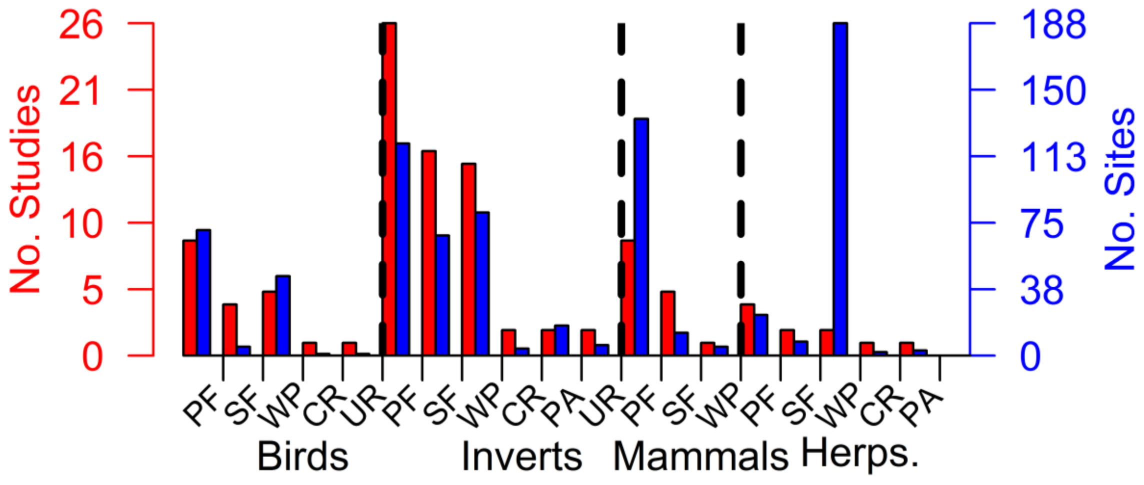

Figure S5. The distribution of sites along gradients of human population density and iNDVI, for each of the major taxonomic groups considered. Different colours refer to the different studies from which the data were sourced. See main text for details of how the environmental gradients were measured.

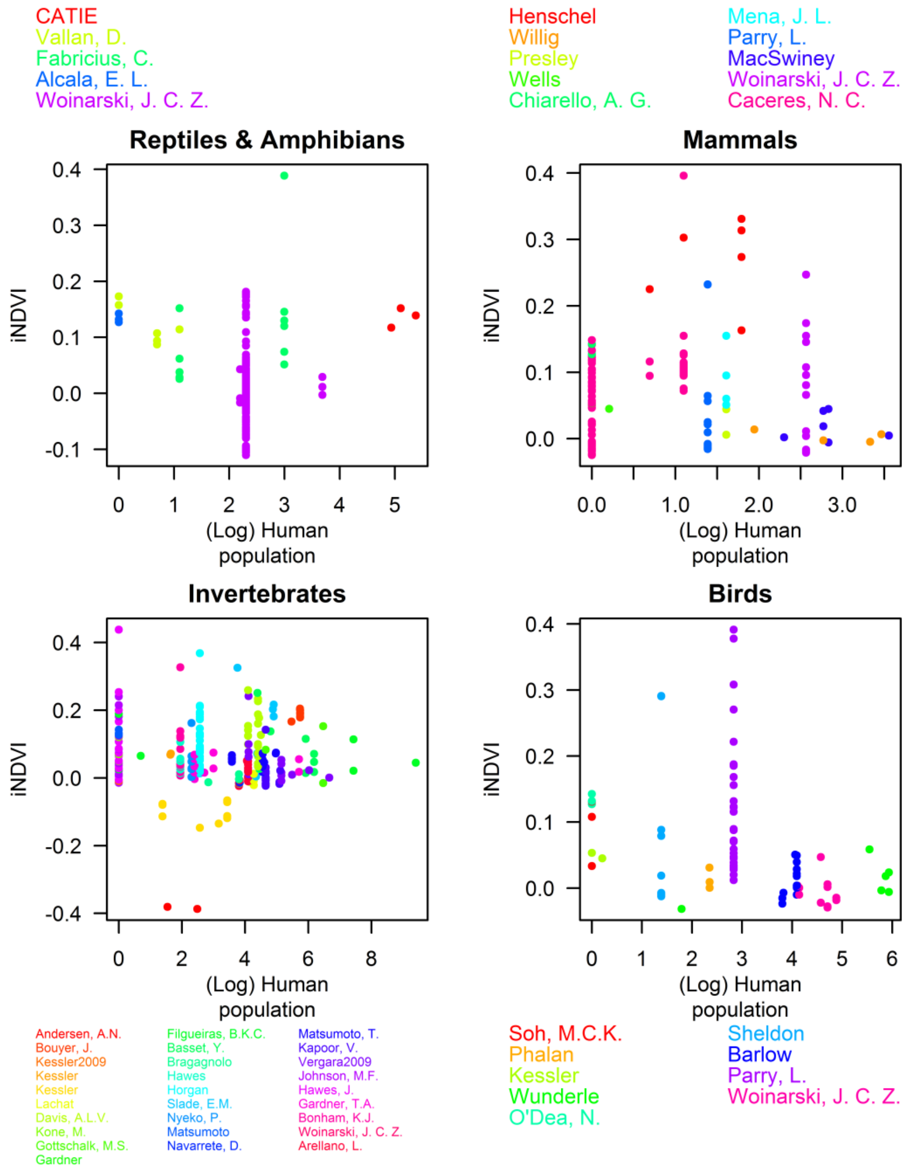

Figure S6. The distribution of sites along gradients of human population density and forest cover, for each of the major taxonomic groups considered. Different colours refer to the different studies from which the data were sourced. See main text for details of how the environmental gradients were measured.

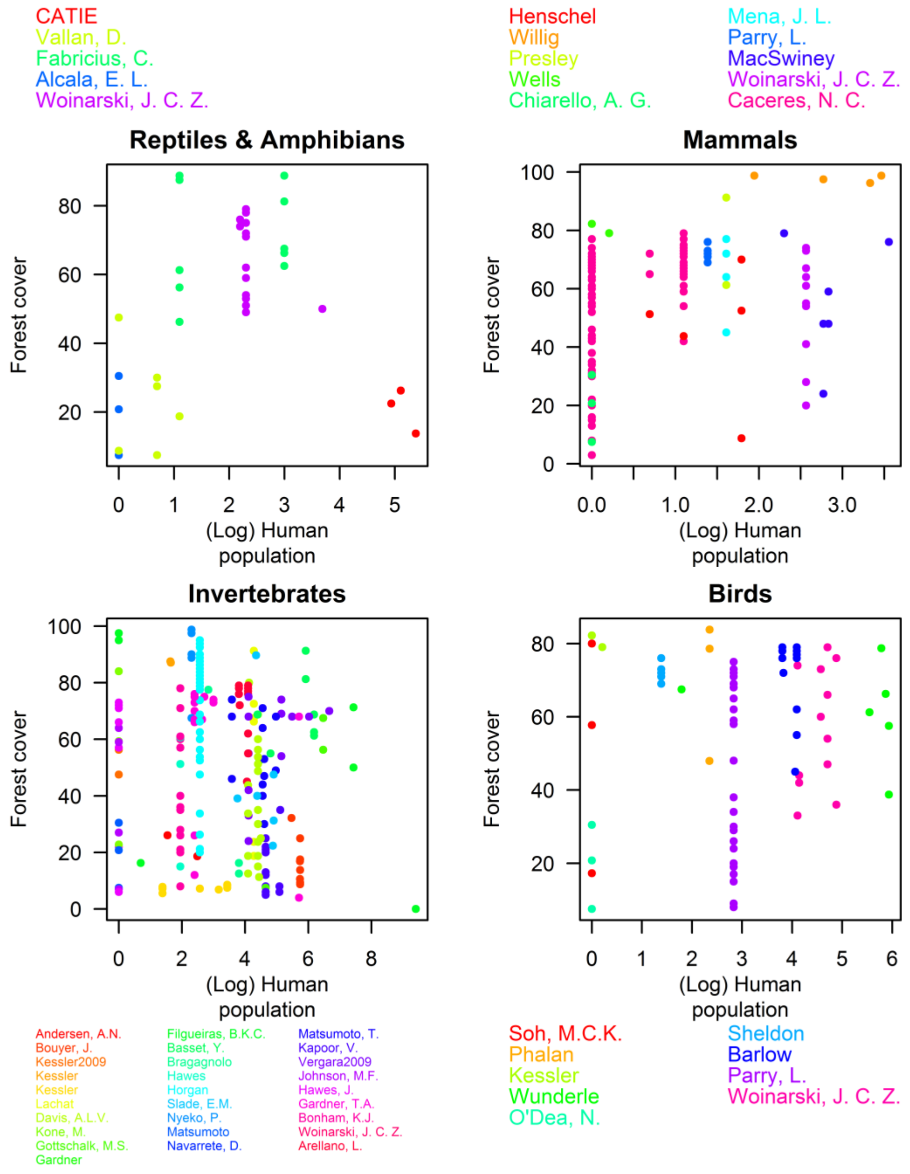

Figure S7. Across the studies from which data were taken, the distribution of P-values testing for spatial autocorrelation in the residuals of the models of species occurrence. Residuals for 17.3% of studies showed significant spatial autocorrelation (shown to the left of the vertical red line).

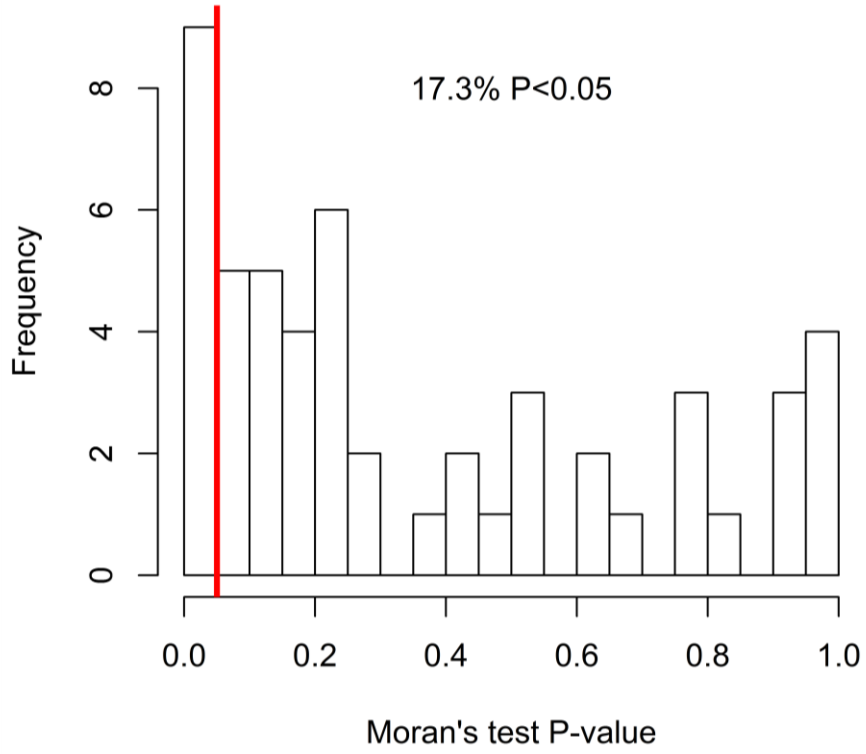

Figure S8. Across the studies from which data were taken, the distribution of P-values testing for spatial autocorrelation in the residuals of the models of species abundance. Residuals for 15.4% of studies showed significant spatial autocorrelation (shown to the left of the vertical red line).

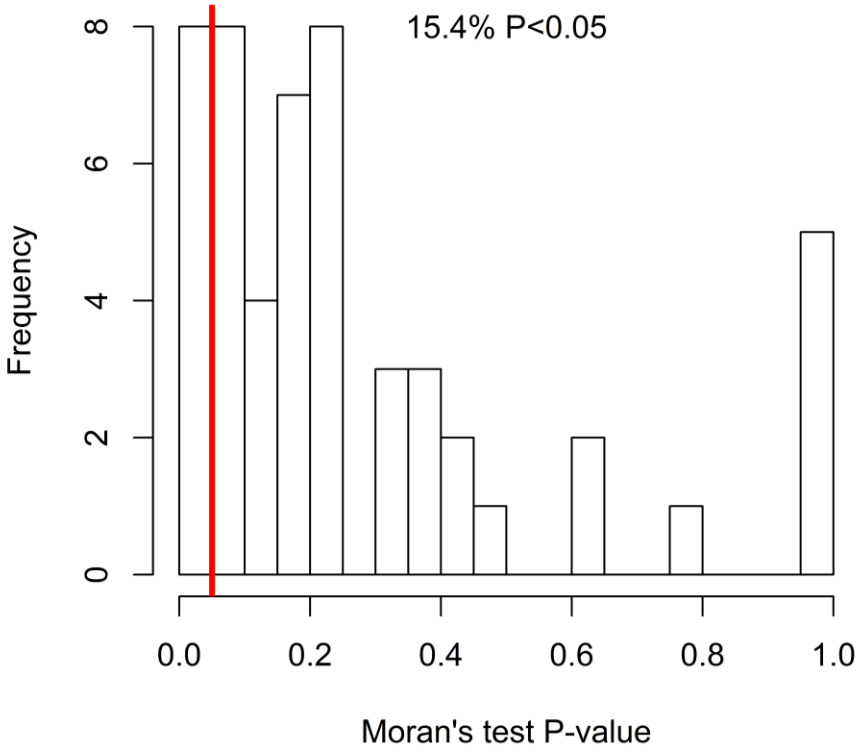

Figure S9 (next page). Response of the probability of occurrence of 3708 taxa in tropical forests to land-use (a), forest cover (b), and the interaction between vegetation removal (iNDVI) and human population density (c-h), for studies for which the residuals of the main models did not show significant spatial autocorrelation. Panel a shows the relative (logit-transformed) probability of occurrence, relative to the probability of occurrence in primary forest; separate responses of forest specialists and habitat generalists are shown by open circles and open triangles respectively; land-use categories considered were: primary forest (PF), secondary forest (SF), plantation forest (WP), cropland (CR), pasture (PA) and urban (UR). Panels b-h show the absolute (untransformed) probabilities of occurrence, with separate panels for forest specialist birds and habitat specialist mammals (spec.), and habitat generalists (gen.). Probability of occurrence was estimated using generalized linear mixed-effects models with a binomial error distribution, fitting site nested within study and taxon as random effects. For the response to forest cover, the average response across all species is shown. Error bars (a), dashed lines (b) and light grey surfaces (c-h) show ± 1 standard error.

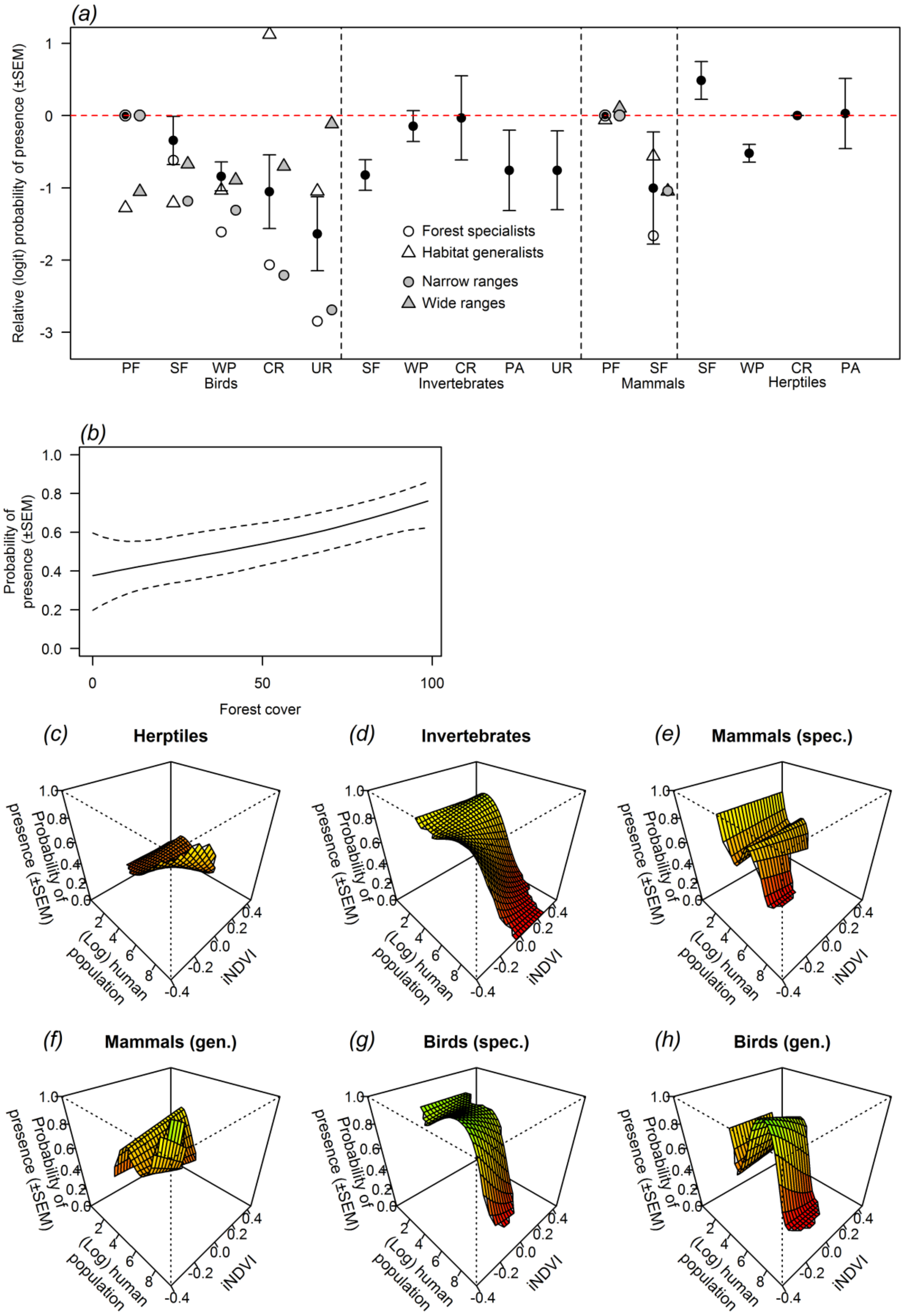
(a)
Relative (logit) probability of presence (±SEM)
Forest specialists
Habitat generalists
Narrow ranges
Wide ranges
PF SF WP CR UR
Birds
SF WP CR PA UR
Invertebrates
PF SF
Mammals
SF WP CR PA
Herptiles
(b)
Probability of presence (±SEM)
Forest cover
(c) Herptiles
(d) Invertebrates
(e) Mammals (spec.)
(f) Mammals (gen.)
(g) Birds (spec.)
(h) Birds (gen.)
Probability of presence (±SEM)
(Log) human population
iNDVI

Figure S10 (next page). Response of the abundance of 3708 taxa in tropical forests to land-use (a), the interaction between human population density and forest cover (b-g), and the interaction between forest cover and vegetation removal (iNDVI; h-m), for studies for which the residuals of the main models did not show significant spatial autocorrelation. Panel a shows the relative (log-transformed) abundance, relative to the abundance in primary forest; separate responses of forest/habitat specialists and habitat generalists are shown by open circles and open triangles respectively; land-use categories considered were: primary forest (PF), secondary forest (SF), plantation forest (WP), cropland (CR), pasture (PA) and urban (UR). Panels b-m show the absolute (log-transformed) abundance, with separate panels for birds and mammals for forest/habitat specialists (spec.) and habitat generalists (gen.). Abundance was estimated using linear mixed-effects models, after log-transforming abundances, fitting site nested within study and taxon as random effects. Error bars (a) and light grey surfaces (b-g) show ± 1 standard error.

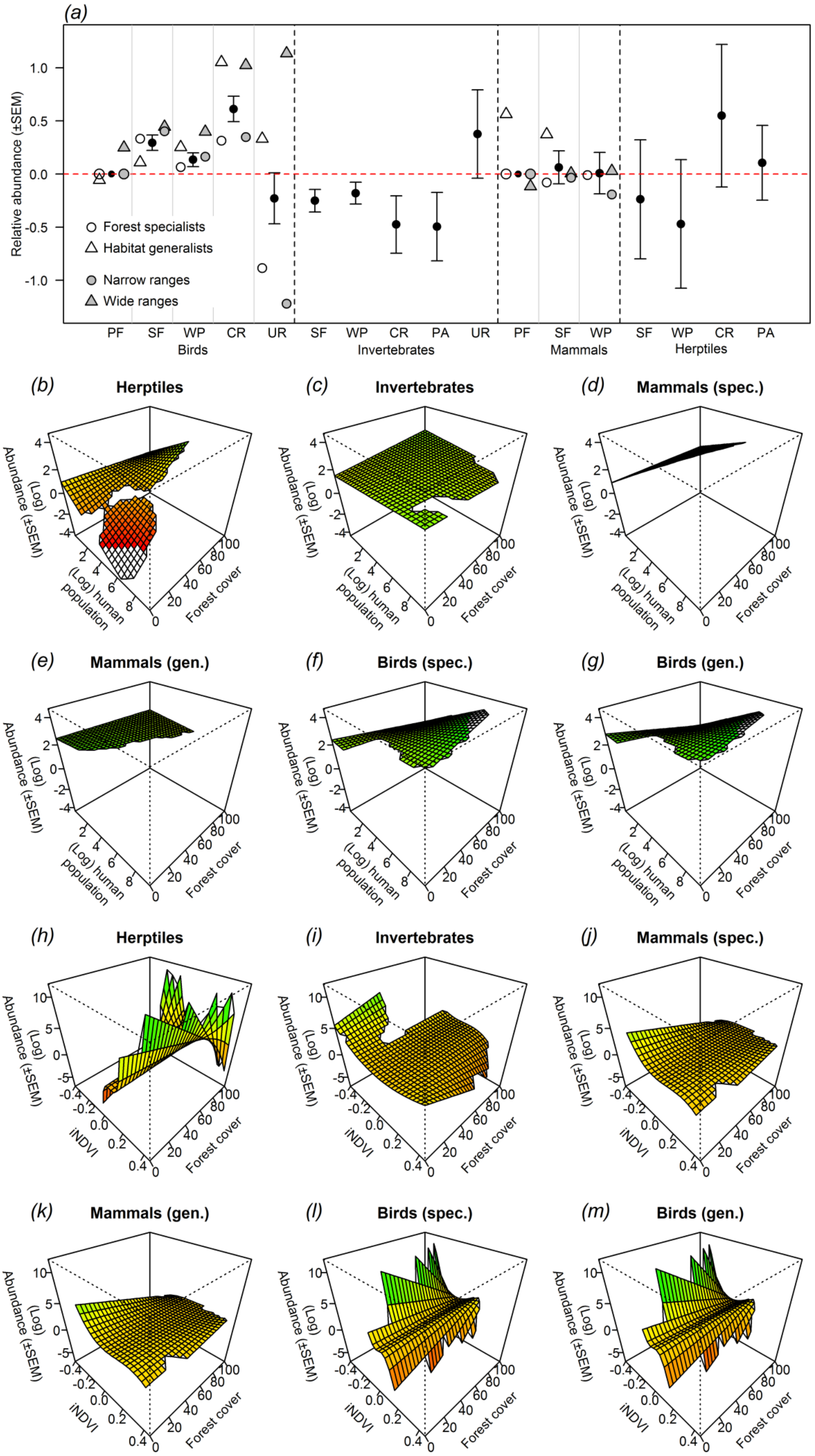
(a)
Relative abundance (±SEM)
1.0
0.5
0.0
-0.5
-1.0
Forest specialists
Habitat generalists
Narrow ranges
Wide ranges
PF SF WP CR UR
Birds
SF WP CR PA UR
Invertebrates
PF SF WP
Mammals
SF WP CR PA
Herptiles
(b) Herptiles
(c) Invertebrates
(d) Mammals (spec.)
(e) Mammals (gen.)
(f) Birds (spec.)
(g) Birds (gen.)
(h) Herptiles
(i) Invertebrates
(j) Mammals (spec.)
(k) Mammals (gen.)
(l) Birds (spec.)
(m) Birds (gen.)
(Log) Abundance (±SEM)
(Log) human population
Forest cover
iNDVI
